# Microplastic induced cascades of multiple cell death pathways: inflammation, immune imbalance, and cancer susceptibility

**DOI:** 10.3389/fpubh.2026.1762893

**Published:** 2026-05-22

**Authors:** Jiarong Du, Mingjie Wang, Chunyan Wang

**Affiliations:** 1 Affiliated Hospital of Inner Mongolia Medical University, Hohhot, Inner Mongolia, China; 2 Department of Endocrinology, Affiliated Hospital of Inner Mongolia Medical University, Hohhot, Inner Mongolia, China; 3 Department of Ophthalmology, Affiliated Hospital of Inner Mongolia Medical University, Hohhot, Inner Mongolia, China

**Keywords:** cancer susceptibility, cell death pathways, immune imbalance, inflammation, microplastic

## Abstract

Microplastic and nanoplastic (M/NP) pollution has evolved from an environmental issue into a pressing threat to human health, with accumulating evidence showing that residue levels in human tissues are rising annually. Beyond their widespread distribution, the toxicity of these particles is critically determined by their physicochemical properties, particularly particle size, specific surface area, and polymer type. Notably, nanoplastics exhibit a high potential for crossing biological barriers, and their formation of biocoronas significantly alters their biological identity and interaction with host cells. This review systematically outlines the physiopathological pathways by which M/NPs damage the human body. We elucidate how M/NPs, following internalization via endocytosis, trigger excessive reactive oxygen species (ROS) generation, leading to organelle stress, genotoxicity, and potential nuclear entry. Crucially, we discuss the cascade network of multiple regulated cell death modalities, including apoptosis, ferroptosis, pyroptosis, and autophagy, as interconnected drivers of tissue injury. Furthermore, we explore the progression from chronic immune activation to immune exhaustion, highlighting how these dysregulated inflammatory responses remodel the tissue microenvironment and potentially promote cancer susceptibility. By synthesizing these mechanisms, this study aims to persuade the reader of the severe health risks posed by M/NPs and provides a theoretical foundation for future mechanistic investigations and human health risk assessments, rather than merely offering strategies for pollution control.

## 1 Background

Microplastics are generally defined as plastic fragments measuring ≤ 5 mm in size. They can be broadly categorized into primary and secondary microplastics and are now recognized as a globally important class of environmental pollutants with highly heterogeneous compositions (1, 2). Primary microplastics refer mainly to small plastic particles or flakes intentionally manufactured for industrial or commercial use, such as microbeads in cosmetics, personal care products, and paints, as well as resin pellets used in plastic production (1, 3). By contrast, secondary microplastics arise predominantly from the progressive fragmentation of larger plastic materials under environmental conditions, including light exposure, temperature fluctuations, mechanical abrasion, and biological activity. Representative examples include particles released from textiles and tires during use, as well as degradation products generated from the long-term weathering of plastic debris in the environment (4, 5). Together, these diverse sources indicate that microplastics are both ubiquitous and difficult to completely intercept during production and consumption, making their continuous release into the environment a major global challenge.

Plastic pollution is now pervasive across both marine and terrestrial ecosystems worldwide. Microplastics have been identified in approximately 1,300 aquatic and terrestrial species, as well as in multiple human tissues and organs (1, 6). It has been estimated that approximately 10–40 million tons of microplastics enter the environment each year, and this amount may double by 2040 if current patterns of plastic production and waste management remain unchanged (1). Modeling studies further suggest that continued accumulation over the next 70–100 years could lead to large-scale and potentially irreversible ecological damage (1). The environmental behavior and ecotoxicity of plastic particles are strongly influenced by their surface physicochemical properties, including roughness, charge, and hydrophobicity (7). Notably, different types of microplastics have been detected in human placenta, meconium, breast milk, blood, and feces, indicating that exposure may occur across multiple life stages, from fetal development to adulthood (2). These findings not only underscore the ubiquity of human exposure but also raise growing concerns regarding potential systemic health effects.

Microplastics can enter the human body through the food chain and accumulate at both tissue and cellular levels (8). In environmental monitoring and toxicological studies, polyethylene (PE), polypropylene (PP), polyethylene terephthalate (PET), polyvinyl chloride (PVC), and polystyrene (PS) are among the most frequently investigated polymer types (9). Of these, PE is widely detected in environmental samples because of its high production volume; PP is commonly associated with food packaging and medical products; and PS is one of the most extensively used model microplastics in toxicological research because of its prevalence in disposable tableware and packaging materials (9, 10). The biodistribution and clearance of microplastics within the body are largely dependent on particle size. Using polystyrene micro-/nanoplastics (PS-MNPs) as an example, particles of different diameters, such as 0.25 μm, 1 μm, and 10 μm, show marked differences in systemic absorption and transport (11). Smaller particles generally exhibit greater absorption efficiency in target organs such as the gut, brain, and lungs. In particular, smaller MNPs, such as 0.25 μm particles, are more readily internalized by cells through endocytosis than larger particles. Consistent with this, significantly greater accumulation of smaller particles has been observed in several short- and medium-term exposure studies using colon cancer cell lines (11). When particle size decreases further to below 10 nm, their behavior has been described as resembling that of gaseous substances, with high permeability that may enable them to cross tissue barriers and directly enter cells (12). This suggests that nanometer-scale, or near-nanoscale, plastic particles may be especially capable of disrupting intracellular homeostasis and triggering a cascade of cytotoxic events.

Against this background, the bioaccessibility and bioaccumulation of microplastics in the human body suggest that they should not be regarded merely as inert foreign particles. Rather, microplastics may trigger multiple forms of regulated cell death, activate or dysregulate inflammatory pathways, and ultimately disturb immune microenvironmental homeostasis. Chronic low-dose exposure may also be closely associated with increased cancer susceptibility. However, a systematic understanding is still lacking regarding the cascade relationships among microplastic-induced cell death programs, such as apoptosis, necroptosis, pyroptosis, and ferroptosis, and how these processes interact with inflammatory amplification, immune imbalance, and tumorigenic risk (13, 14).

**Figure 1:**
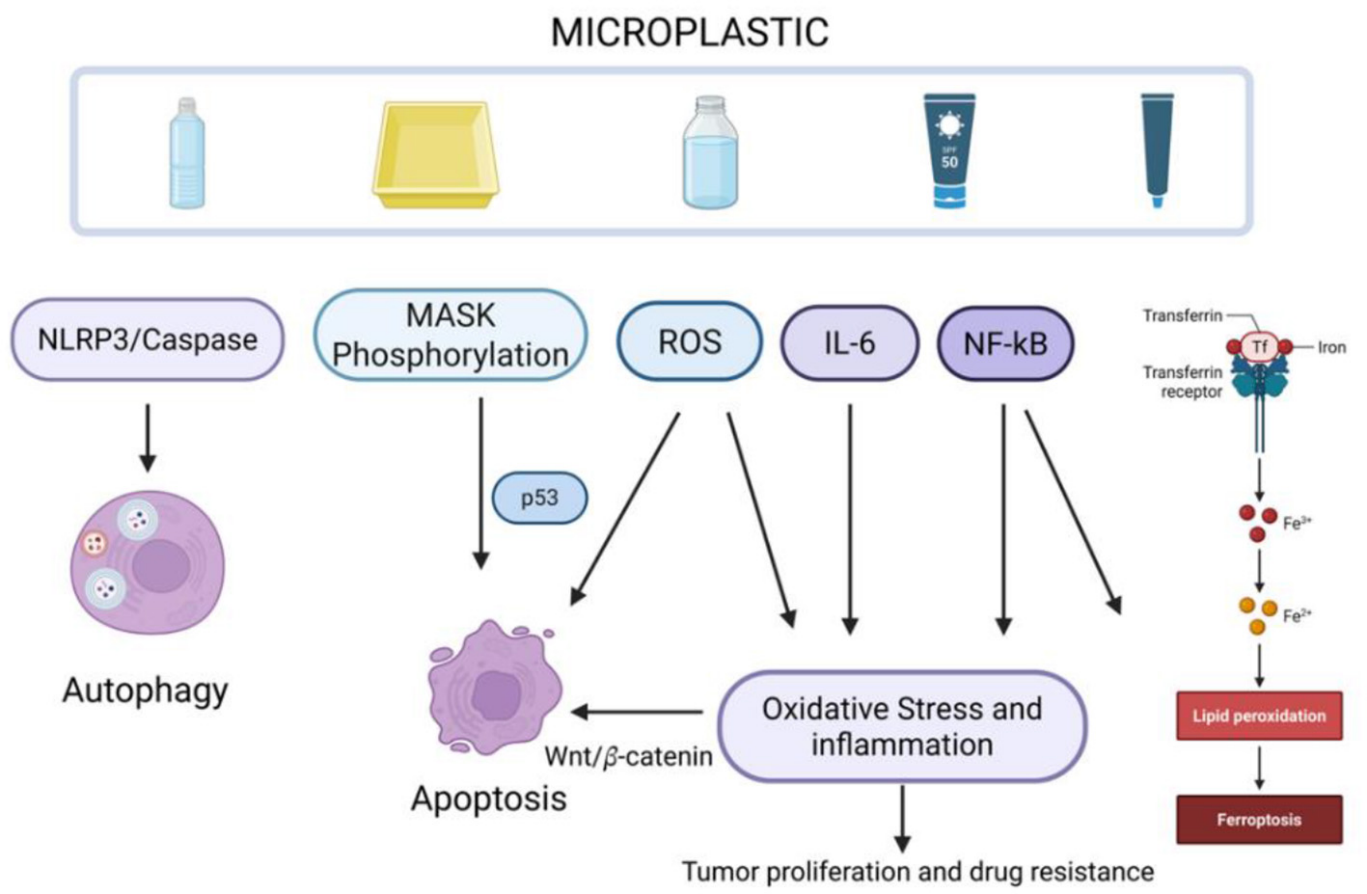
Microplastic-mediated regulatory network of cell death, inflammation, and immune dysregulation.

Given the accumulating evidence that microplastic residues are widespread in human tissues and appear to be increasing annually, it is essential to clarify the pathophysiological mechanisms through which these pollutants harm the human body. Accordingly, this review focuses on the central theme of microplastic-induced cascades of multiple cell death pathways. We first summarize microplastic cytotoxicity and the major forms of regulated cell death, and then systematically integrate the available experimental and epidemiological evidence to delineate the relationships between exposure characteristics and biological outcomes. We further discuss how microplastics may promote the accumulation of cellular damage and initiate carcinogenesis-related processes by inducing inflammatory responses, disrupting immune homeostasis, and remodeling the tissue microenvironment (Figure 1), thereby increasing cancer susceptibility. Through this synthesis, we aim to highlight the potential health risks posed by microplastic pollution and to provide a theoretical basis for future mechanistic studies and health risk assessment.

## 2 Cellular uptake and bioaccumulation

### 2.1 Tissue distribution and size-dependent accumulation

Particle size is a key determinant of the cellular uptake efficiency and early toxic potential of microplastics (MPs) (15). Microplastic contamination has now been widely detected in human biological matrices, including blood (16), placenta (17), and liver (18), confirming that these particles can enter the systemic circulation. Studies have shown that human cells are capable of internalizing nanoplastics (NPLs) (19). Owing to their extremely small size, NPLs can be absorbed and translocated to multiple organs and tissues, resulting in intracellular bioaccumulation and posing potential risks to human health (16, 20, 21). Compared with larger microplastics (MPLs), NPLs have a greater ability to cross cellular membrane barriers and generally exhibit higher bioavailability, stronger toxicity, and a greater capacity to induce oxidative stress (15, 22–24). *In vitro* studies have further demonstrated substantial bioaccumulation of polystyrene nanoplastics (PS-NPLs) ranging from 50 to 500 nm in lung epithelial cells, intestinal cells, intestinal organoids, and macrophages (25–27).

Animal studies provide further evidence for the *in vivo* transport and distribution of micro- and nanoplastics (MNPs). PS and PE particles of various sizes have been shown to accumulate in metabolically active organs, particularly the intestine and liver, in fish species such as zebrafish and crucian carp (28). Notably, nanoscale particles exhibit greater tissue penetration capacity. After zebrafish larvae were exposed to PS nanoparticles smaller than 100 nm, these particles were able to cross the intestinal barrier, translocate from the intestinal lumen to the liver, and even pass through the blood–brain barrier into brain tissue (29). However, the relationship between accumulation burden and toxicity is not always linear. In a study of red tilapia exposed to PS N/MPs of three different sizes (0.3 μm, 5 μm, and 70–90 μm), larger particles showed higher levels of tissue accumulation, whereas toxic effects did not display a simple inverse relationship with particle size. This finding suggests that the link between bioaccumulation and toxicity is more complex than particle size alone would predict (30).

### 2.2 Biocorona formation and physicochemical evolution

During their passage through biological fluids, the physicochemical properties of NPLs can undergo substantial changes. Owing to their high surface-area-to-volume ratio, surface charge, and polymer composition, NPLs readily interact with lipids, nucleic acids, and proteins, leading to the formation of a “biocorona” on their surface (31, 32). These biomolecules, derived mainly from extracellular polymers or intracellular components, confer a new “biological identity” on nanoplastics, thereby shaping their toxicological behavior (31). As the key interface between NPLs and biological systems, the biocorona directly affects their biodistribution, translocation, cellular internalization, and clearance (33). In addition, the biocorona can modify toxicity by altering the toxicokinetic and toxicodynamic profiles of NPLs, with effects that may be synergistic, antagonistic, or neutral (31).

### 2.3 Cellular uptake pathways and membrane damage

Microplastics can enter cells through several distinct pathways, including endocytosis, passive diffusion, and direct membrane translocation (34–36). In addition to these uptake mechanisms, the direct physical damage that particles inflict on the cell membrane also warrants attention. PE microplastics can adhere to and insert into the lipid bilayer, promoting pore formation and causing concentration-dependent structural damage to phospholipid components such as dipalmitoylphosphatidylcholine (DPPC) (37). Moreover, PE particles may destabilize lipid membranes through mechanical stretching, thereby compromising membrane barrier integrity and facilitating the entry of harmful substances (38).

### 2.4 Intracellular trafficking fate and autophagy disruption

After entering the cell, microplastics are typically first sequestered within early endosomes. They may then be removed through exocytosis, escape from endosomal compartments, or, in some cases, be cleared by passive diffusion (34). However, under conditions of persistent intracellular accumulation, nanoplastic-containing vesicles can mature from late endosomes into autophagosomes and subsequently fuse with lysosomes to form autolysosomes. Because lysosomal enzymes are unable to effectively degrade nanoplastics, this process can impair autophagic flux and cause lysosomal damage, leading to the leakage of lytic enzymes into the cytoplasm. Released nanoplastics and their associated protein coronas may further promote the formation of new protein aggregates, thereby amplifying cellular injury (39).

## 3 Microplastic-induced oxidative stress and inflammation

### 3.1 Oxidative stress: a central mechanism of microplastic toxicity

Oxidative stress is widely recognized as one of the central mechanisms underlying micro- and nanoplastic (MNP)-induced toxicity (40). Exposure to MNPs has been shown to markedly increase the production of reactive oxygen species (ROS), often accompanied by elevated levels of lipid peroxidation (41, 42). The severity of these effects is strongly influenced by physicochemical properties such as particle size and polymer type. Smaller particles, particularly nanoplastics below 50 nm, are more readily internalized by cells and can rapidly induce intracellular oxidative stress. In some cases, they may even directly alter the structure and function of antioxidant enzymes, including superoxide dismutase (SOD) and catalase (CAT) (43, 44). In addition, polymer type contributes to differences in transport and toxicity profiles. For example, PE has been reported to cross the intestinal epithelium more efficiently than polystyrene (PS) (9). Moreover, although PE showed no significant effects on the viability of T98G and HeLa cells at certain doses, it selectively increased ROS levels in Caco-2 and A549 cells (45).

Persistent oxidative stress further weakens the cellular antioxidant defense system, as reflected by reduced CAT and glutathione activity together with increased malondialdehyde levels (46, 47). As an upstream regulator of the cell cycle, energy metabolism, and apoptosis, excessive ROS accumulation not only disrupts cellular homeostasis but also promotes DNA damage and genetic mutations, thereby potentially increasing susceptibility to malignant transformation (42, 44, 48).

### 3.2 Inflammatory activation pathways and immune responses

Disruption of cellular homeostasis by MNPs is often accompanied by a pronounced inflammatory response. Current evidence suggests that MNP-induced pro-inflammatory signaling is mediated primarily through the TLR4/p38 axis and involves dose-dependent activation of pro-inflammatory cytokines via the MAPK/NF-κB pathway (49–51). This immunostimulatory effect appears to depend on both particle size and polymer type, with smaller nanoplastics generally showing greater immunoreactivity than larger particles. For example, PS-NPLs markedly increase the levels of TNF-α, IL-6, and IFN-γ in trophoblast cells and macrophages such as RAW 264.7 cells (50, 52, 53). Similarly, nanoscale polyamide (PA) exerts stronger pro-inflammatory and cytotoxic effects on bronchial epithelial cells than its microsized counterparts (54).

Evidence from both *in vivo* experiments and clinical studies further supports these mechanisms. Inhalation studies in mice have shown that PS and PP microplastics can induce pulmonary inflammation through activation of the NLRP3 inflammasome pathway (55). Clinical observations have also revealed that human carotid plaques containing PE microplastics exhibit significantly elevated levels of pro-inflammatory cytokines, including IL-18, IL-1β, and TNF-α, together with marked leukocyte infiltration, as indicated by CD3 and CD68 staining (56). Notably, larger PP microplastics (20–200 μm) have also been reported to stimulate inflammatory cytokine production, suggesting that pro-inflammatory activity is a general biological feature of MNP exposure rather than a property limited to nanoscale particles (57, 58).

### 3.3 Coupling of oxidative stress and inflammation and their systemic pathological consequences

The interplay between oxidative stress and inflammation is a key feature of the systemic toxicity induced by MNPs. Evidence suggests that MNP exposure can cause substantial tissue injury and functional impairment by simultaneously downregulating placental tight junction proteins, suppressing the Nrf2/HO-1 antioxidant pathway, and upregulating pro-inflammatory cytokines (59–61). In addition, environmental factors such as ultraviolet (UV) oxidation may further enhance the ability of microplastics to induce intracellular ROS production by promoting the release of oxidized fragments (62).

Together, these multi-pathway toxic effects can ultimately lead to widespread systemic damage. Through the combined actions of oxidative stress and inflammation, MNP exposure has been linked to DNA damage, metabolic dysregulation, and endocrine disruption (63). In murine models, long-term exposure has also been implicated in the development of non-alcoholic fatty liver disease, potentially through alterations in gut microbiota composition and the exacerbation of liver fibrosis (64).

## 4 Microplastic-mediated multiple cell death modalities

### 4.1 Cascade activation and crosstalk networks of cell death

In the context of microplastic-induced toxicity, apoptosis, pyroptosis, and ferroptosis do not occur as isolated events; rather, they form an interconnected and mutually reinforcing network of cell death pathways (65). Microplastics can activate these programs by inducing excessive ROS production, disrupting mitochondrial and endoplasmic reticulum (ER) homeostasis, and modulating key signaling pathways. This complex cell death network not only aggravates local tissue injury but also promotes inflammatory responses and immune dysfunction, thereby posing a broader systemic threat to host health (14).

### 4.2 ROS-mediated organelle stress and apoptotic pathways

Microplastic-induced apoptosis is closely linked to ROS-driven organelle stress. Evidence indicates that MPs activate the ER stress-associated GRP78/IRE1α/JNK axis, thereby inducing placental cell apoptosis and impairing placental function (59). In the reproductive system, MP exposure has been associated with reduced sperm motility as well as atrophy and apoptosis of germ cells at multiple developmental stages (42, 60). In particular, exposure to 5 μm polystyrene microplastics (PS-MPs) has been shown to markedly reproduce apoptotic phenotypes in testicular tissue (60). In pulmonary tissue, PS-MP exposure increases the Bax/Bcl-2 ratio and upregulates caspase family proteins, thereby promoting the apoptotic cascade through activation of MAPK signaling pathways, including p38, ERK, and JNK (47). At the molecular level, MPs can induce apoptosis through the ROS–p53 axis and establish a positive feedback loop between apoptosis and DNA damage driven by mitochondrial dysfunction, further amplifying cellular injury (66, 67). In addition, aged microplastics have been reported to enhance mitochondria-mediated apoptotic signaling (68), whereas apoptosis in ovarian granulosa cells appears to be closely associated with activation of the Wnt/β-catenin pathway (69).

### 4.3 Lipid peroxidation-driven ferroptosis

Ferroptosis is a form of regulated cell death characterized by intracellular iron overload and lipid peroxidation (70). Emerging evidence indicates that microplastics can trigger ferroptosis in multiple tissues, particularly in the intestine and liver, and that this process is closely linked to oxidative stress, inflammatory signaling, and disrupted iron homeostasis. In intestinal tissue, microplastic exposure has been shown to promote lipid peroxidation and activate NF-κB signaling, thereby inducing ferroptosis accompanied by enhanced apoptosis (71). Similar ferroptosis-related alterations have also been reported in liver models (72). Mechanistically, microplastic-induced ferroptosis appears to involve coordinated disruption of antioxidant defense pathways, mitochondrial redox balance, and iron metabolism. In avian models, microplastic exposure promoted the accumulation of glutamine and glutamate, suppressed the Nrf2–Keap1–HO-1/NQO1 antioxidant defense axis, and triggered autophagy-dependent ferroptosis, ultimately leading to severe tissue injury (73).

Consistent with this, both *in vitro* and *in vivo* studies have shown that MPs can aggravate COPD-related inflammatory responses by enhancing mitochondria-derived ROS (mito-ROS)-mediated autophagy-dependent ferroptosis (74). During microplastic-induced liver injury, ferroptosis has been observed together with strong oxidative stress, inflammation, and pyroptosis, indicating extensive crosstalk among multiple cell death programs (72). These changes are accompanied by activation of transferrin receptor (TFRC), suppression of ferritin heavy chain 1 (FTH1), inhibition of the cystine/glutamate antiporter system Xc– and glutathione peroxidase 4 (GPX4), and dysregulation of ACSL4, collectively reflecting profound disturbance of iron handling and lipid peroxide detoxification (72).

Particle size may further modulate the ferroptotic response. Smaller nanosized polystyrene particles (100–500 nm) are more readily internalized by epithelial cells and induce more pronounced lipid peroxidation, mitochondrial membrane potential loss, and downregulation of key ferroptosis-related proteins such as SLC7A11 and GPX4 (72). Evidence from fish and other aquatic animal models likewise suggests that microplastics can alter metal ion distribution, increase free iron and ROS levels, and thereby regulate ferroptosis, contributing to intestinal iron dyshomeostasis, inflammatory injury, and organ damage (75).

Taken together, these findings suggest that ferroptosis is an important component of microplastic-induced toxicity. Rather than acting as an isolated event, ferroptosis appears to function within a broader network of oxidative stress, inflammation, autophagy, and other regulated cell death pathways, thereby amplifying tissue injury under microplastic exposure conditions.

### 4.4 Pyroptosis and autophagy as synergistic amplifiers of toxicity

**Figure 2:**
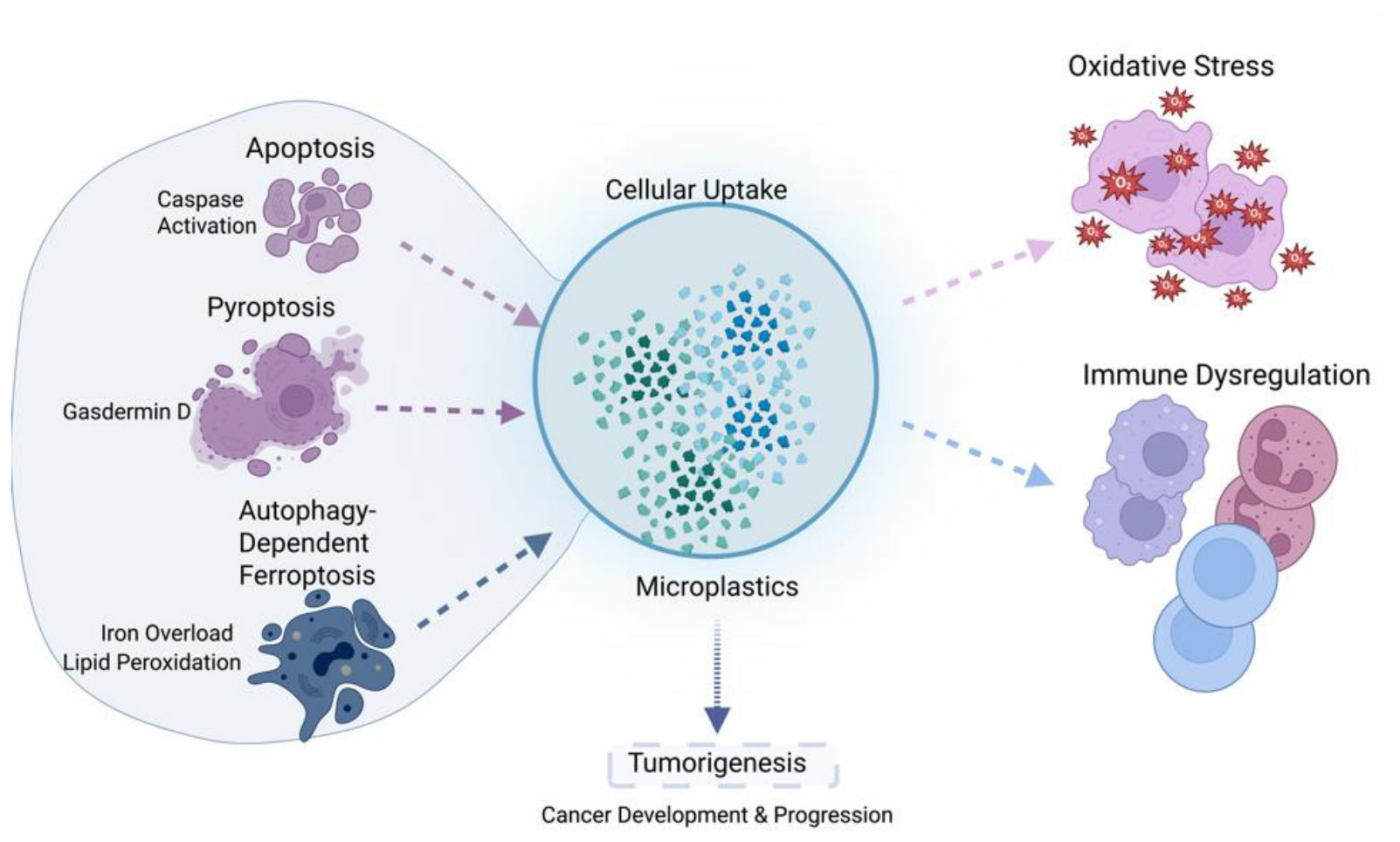
Microplastic-induced multiple cell death cascades and their pathological consequences.

Pyroptosis and autophagy play important regulatory and amplifying roles in microplastic-induced cellular injury. Oxidative stress promotes assembly of the NLRP3 inflammasome and activation of caspase-1, thereby driving the maturation and release of pro-inflammatory cytokines (76, 77). In ovarian granulosa cells, PS-MPs have been shown to induce pyroptosis through the NLRP3/caspase-1 pathway, with apparent synergy with apoptosis (77). Studies in zebrafish further indicate a clear size-dependent effect, with 0.5 μm particles being more potent than 5 μm particles in reducing macrophage abundance and increasing apoptosis (78, 79). The interplay between autophagy and apoptosis represents another important feature of microplastic toxicity. Exposure to PS-MPs can promote autophagy by upregulating DDIT4 and inhibiting the mTOR pathway (69, 80). In renal epithelial cells, microplastics simultaneously induce oxidative stress, autophagy, inflammation, and apoptosis, ultimately leading to renal tubular injury and metabolic dysregulation (81–83). This process is commonly associated with reduced phosphorylation within the PI3K/Akt/mTOR pathway, together with canonical markers of autophagy activation, including increased LC3 expression and p62 degradation (47). As summarized in Figure 2, microplastic exposure triggers a cascade of multiple regulated cell death pathways, which further interact with oxidative stress and immune dysregulation, ultimately contributing to tumor-promoting pathological changes.

## 5 MNP-induced immune system disorders

### 5.1 Initial recognition and response of innate immunity

The immune system is a highly coordinated defense network composed of multiple cellular and molecular components. During the early stages of exposure to MNPs, innate immunity plays a central protective role through tissue barriers, pattern recognition receptors, and phagocytic processes (84). Macrophages and neutrophils are rapidly recruited to sites of injury, where they release inflammatory mediators such as interleukin-1β (IL-1β) and tumor necrosis factor-α (TNF-α) in response to cellular stress and tissue damage (85). In addition, through interactions with the host microbiota, MNPs may promote persistent activation of innate immune responses, leading to the sustained release of pro-inflammatory cytokines and chemokines (86).

### 5.2 Physicochemical property-mediated barrier damage and combined toxicity

The distinctive physicochemical properties of MNPs, including high hydrophobicity, large specific surface area, and surface charge, make them highly prone to adsorbing heavy metals and persistent organic pollutants from the environment, thereby acting as composite carriers with enhanced toxicity (87–89). In fish models, polyethylene microplastics (PE-MPs) have been shown to markedly disrupt mucosal immunity. For example, exposure of adult zebrafish to PE-MPs reduced goblet cell coverage and caused a dose-dependent decrease in complement C3 and C4 levels, accompanied by upregulation of genes associated with mucosal immunoglobulin responses (90). Similar findings have been reported in grass carp, common carp, and yellow catfish, suggesting that MNP exposure may broadly disturb local immune network function across aquatic species (91–93). Tissue accumulation of MNPs not only causes direct physical injury and chemical toxicity but may also impair immune function at the bioenergetic level by interfering with nutrient absorption and altering energy allocation (94).

### 5.3 Systemic distribution, cellular internalization, and immune polarization

Following systemic distribution through the circulation, MNPs can penetrate and accumulate in lymphoid tissues and secondary immune organs, where they disrupt cellular homeostasis through endocytosis and tissue infiltration (95, 96). Because they are resistant to biodegradation, MNPs may persist within immune cells for prolonged periods, interfering with normal cellular functions through sustained physical and biochemical stress (97). In mammalian models, synthetic polymer microplastics have been shown to induce immune lineage remodeling, characterized by upregulation of immune-related genes and increased production of Th2-type cytokines and immunoglobulins, indicating a shift toward immune polarization (85). This immunotoxicity is often accompanied by altered metabolic profiles, increased apoptosis, and dysfunction of immune-related organs such as the liver and intestine (98).

### 5.4 Progression from chronic activation to immune exhaustion

Long-term or high-intensity exposure to microplastics may drive a pathological transition from defensive immune activation to functional exhaustion. In humans, the persistent presence of microplastics has been proposed to contribute to chronic inflammation, which may in turn be associated with growth retardation, reduced reproductive capacity, and systemic immune impairment (59, 99–104). Long-term observations in aquatic organisms further illustrate this progression. During the early phase of exposure, macrophages and T cells are actively recruited and lysosomal activity is enhanced; however, with prolonged exposure, rates of immune cell apoptosis increase significantly, whereas lysosomal degradative capacity and complement levels gradually decline (98). This shift from initial immune activation to immune exhaustion and dysregulation highlights an important mechanism by which chronic microplastic exposure may progressively undermine the organism's immune defense system (98).

## 6 Potential role of microplastics in tumor promotion and progression

### 6.1 Carcinogenic potential and vector effects of microplastics

In recent years, the potential association between MNP exposure and carcinogenesis has attracted increasing academic attention (11). Although microplastics may not themselves be direct carcinogens, they can act as vectors for toxic substances, including polycyclic aromatic hydrocarbons (PAHs), heavy metals, and persistent organic pollutants (POPs) (2, 105, 106). The accumulation and subsequent release of these hazardous compounds in the body can disrupt normal cellular function and exacerbate oxidative stress and DNA damage. Together, these effects may promote gene mutations, abnormal cell proliferation, and immune dysfunction, thereby contributing to malignant transformation (2, 61).

### 6.2 Nuclear translocation, genotoxicity, and chromosomal abnormalities

The biological effects of microplastics are closely related to their intracellular localization. Evidence suggests that histone deacetylase 6 (HDAC6)-mediated retrograde transport facilitates the accumulation of PS-NPLs in the perinuclear region (39, 107). Notably, PS-NPLs smaller than 50 nm may directly enter the nucleus through nuclear pore complexes (108), whereas larger nanoplastics may rely on adsorbed protein coronas to facilitate nuclear translocation (31, 109).

Once inside the nucleus, microplastics may induce substantial genotoxic effects. During interphase, they can interfere with DNA replication and transcription (110), whereas during mitosis, they may contribute to chromosomal abnormalities (12). These intranuclear interactions are closely associated with genomic instability and structural chromosomal damage (111, 112). Prolonged exposure may therefore increase the risk of severe malignancies, including lung cancer, hematologic cancers, breast cancer, and prostate cancer (113).

### 6.3 Remodeling of the tumor microenvironment and chronic inflammatory drivers

MNP exposure may promote tumorigenesis not only by directly enhancing tumor cell proliferation but also by reshaping inflammatory and immune processes involved in tumor initiation and progression (114–116). Inhaled or ingested microplastics may contribute to tumor microenvironment (TME) formation and the establishment of an immunosuppressive state by inducing cytotoxicity, chronic inflammation, and endocrine disruption, thereby facilitating the progression from chronic inflammation to cancer (86, 117). PE-MPs have been reported to induce epithelial–mesenchymal transition (EMT), cellular senescence, and epigenetic dysregulation, including altered expression of microRNAs and DNA methyltransferases (45). Because microplastics can be distributed systemically through the circulation, they may also affect distant tissues. After entering the bone marrow, they may disrupt normal hematopoiesis and promote abnormal proliferation and differentiation, thereby increasing the risk of hematologic malignancies such as leukemia and lymphoma (118, 119). In the gut, microplastics can impair the mucosal barrier, disrupt interactions between the microbiota and epithelial cells, weaken mucosal defenses, and sustain chronic inflammation, collectively increasing the risk of colorectal cancer (CRC) (80, 120).

### 6.4 Promotion of metastasis, metabolic remodeling, and chemoresistance

Particle size appears to be a major determinant of the pro-tumorigenic effects of microplastics. Compared with larger particles, smaller microplastics, such as 0.25 μm particles, more strongly enhance cell motility and therefore may have greater metastatic potential (11).

MNPs may further increase tumor aggressiveness by remodeling cellular metabolism and signaling pathways. For example, in CRC cells, microplastic exposure has been shown to enhance lipid uptake through activation of NF-κB signaling. The resulting lipid accumulation suppresses the NLRP3/caspase-1/GSDMD axis, thereby promoting tumor cell survival and drug resistance through inhibition of pyroptosis (121). In skin cancer cells, microplastics have been reported to increase mitochondrial ROS levels, triggering opening of the mitochondrial permeability transition pore (mPTP), release of mitochondrial DNA (mtDNA) into the cytoplasm, and subsequent activation of NLRP3, ultimately promoting cancer cell proliferation (122). Animal studies further support these findings, showing that microplastic exposure not only increases the incidence of CRC but also significantly enhances tumor resistance to platinum-based chemotherapeutic agents such as oxaliplatin (80, 121).

## 7 Discussion

### 7.1 Evidence from *in vitro* studies

Current mechanistic understanding of MNP toxicity is based largely on *in vitro* studies. These studies consistently show that MNPs can induce oxidative stress, mitochondrial and endoplasmic reticulum dysfunction, lysosomal damage, DNA injury, and multiple forms of regulated cell death, including apoptosis, pyroptosis, ferroptosis, and autophagy-related disruption. *In vitro* systems have also been particularly useful for identifying intracellular signaling pathways, such as ROS–p53 activation, NLRP3 inflammasome assembly, and PI3K/Akt/mTOR dysregulation.

However, the strength of these findings should not be overstated. Many studies use pristine, spherical polystyrene particles at concentrations that may exceed realistic human exposure levels, and simplified monoculture models do not capture tissue complexity, immune interactions, or chronic low-dose exposure conditions. Thus, *in vitro* evidence strongly supports biological plausibility, but it cannot by itself establish human pathological risk.

Nevertheless, these studies provide important mechanistic support. For example, PS microplastics have been shown to increase lysosomal membrane permeability and ROS production in human intestinal cells (123), while PE microplastics reduce viability and enhance oxidative stress in colorectal cell lines (124). Similar *in vitro* evidence has been reported in reproductive and neural cell models, where MNP exposure disrupts membrane integrity, mitochondrial function, inflammatory signaling, and cell survivall (115, 125–140).

### 7.2 Evidence from animal models

Animal studies extend these findings to the organismal level and provide evidence that MNPs can distribute to the intestine, liver, brain, reproductive organs, and immune-related tissues. Across diverse models, exposure has been associated with oxidative damage, inflammatory activation, metabolic disturbance, reproductive toxicity, and immune dysfunction, supporting the view that MNPs are biologically active rather than inert particles.

At the same time, important limitations remain. Experimental designs vary widely in exposure route, particle type, particle size, and dose, and many studies rely on acute or high-dose paradigms that may not reflect environmentally relevant human exposure. Species differences in physiology, immune regulation, and xenobiotic handling further complicate extrapolation to humans. Animal studies therefore provide strong support for hazard identification, but not definitive evidence of human disease causality.

Even so, *in vivo* findings are notable. Microplastic exposure has been linked to intestinal inflammation, oxidative stress, microbiota disruption, and altered metabolic profiles in zebrafish (141). Smaller particles often show greater biological reactivity than larger particles (142), and activation of MAPK signaling and lipid peroxidation has been documented across aquatic and mammalian models (42, 143–147). Reproductive toxicity, particularly reduced sperm quality and motility, has also been repeatedly observed (148).

### 7.3 Evidence from human exposure studies

Human studies have confirmed the presence of microplastics in blood, placenta, breast milk, feces, sputum, lung tissue, vascular tissue, and other biological matrices, demonstrating that real-world exposure is widespread and that internalization occurs in humans. These findings provide essential translational relevance and support the biological plausibility of systemic exposure.

However, the current human evidence is still largely descriptive rather than causal. Most studies focus on particle detection and characterization, while relatively few assess clinical outcomes within robust epidemiological frameworks. Direct evidence linking MNP exposure to cancer incidence, progression, or mortality in humans is still lacking. As a result, the proposed relationship between MNP exposure and carcinogenesis remains mechanistically plausible but epidemiologically unproven.

Available human data do suggest possible systemic relevance. Microplastics have been detected in the blood of most participants in some cohorts (149), and their presence has been associated with altered coagulation and inflammatory biomarkers (150). Detection in brain and lung tissues further raises concern regarding barrier penetration and organ-specific effects (61, 151). Still, tissue detection alone demonstrates exposure, not causation, and this distinction is critical when interpreting potential links to chronic disease and cancer.

### 7.4 Current limitations and future directions

Several major limitations continue to constrain the field. First, substantial heterogeneity in particle size, shape, polymer type, surface chemistry, aging status, and exposure design limits comparability across studies. Second, many experimental models rely on pristine polystyrene particles, whereas environmental microplastics are chemically heterogeneous, weathered, and often carry additives, pollutants, or biocoronas. Third, exposure doses used *in vitro* and *in vivo* often exceed estimated human exposure levels, making dose relevance uncertain.

A further limitation is the lack of long-term human studies with reliable exposure assessment and validated health endpoints. At present, the evidence linking MNP exposure to cancer is predominantly mechanistic and indirect. Moreover, key questions remain unresolved regarding absorption, tissue retention, clearance, biodegradation, and interactions with host factors such as the microbiota, diet, inflammation, and genetic susceptibility.

Importantly, the current literature often conflates hazard with risk. Experimental evidence clearly shows that MNPs can induce oxidative stress, inflammation, organelle dysfunction, and cell death under specific conditions, but whether these effects translate into clinically meaningful disease under realistic human exposure scenarios remains uncertain. Future research should therefore prioritize environmentally relevant particles, standardized exposure characterization, chronic low-dose models, advanced organoid and co-culture systems, multi-omics approaches, and prospective human cohorts.

Given the continued global rise in plastic production and the widespread detection of microplastics in the environment and human tissues (152–156) the ecological and public health relevance of this issue is undeniable. Reducing plastic pollution will require coordinated action in source control, waste management, recycling, and safer material development. At the same time, stronger mechanistic, translational, and epidemiological evidence is needed to define the true health significance of chronic MNP exposure and to clarify whether its proposed links to cancer and other chronic diseases can be substantiated in humans.
